# Within-Trial Persistence of Learned Behavior as a Dissociable Behavioral Component in Hippocampus-Dependent Memory Tasks: A Potential Postlearning Role of Immature Neurons in the Adult Dentate Gyrus

**DOI:** 10.1523/ENEURO.0195-21.2021

**Published:** 2021-08-06

**Authors:** Alessandro Luchetti, Takuma Yamaguchi (山口拓馬), Masato Uemura, Glen Yovianto, Luka Čulig, Ming Yang, Wei Zhou, Franziska Oschmann, MinFeng Lua, Ayumu Tashiro (田代 歩)

**Affiliations:** 1 School of Biological Sciences, Nanyang Technological University, Singapore 308232; 2 Kavli Institute for Systems Neuroscience, Norwegian University of Science and Technology, Trondheim 7491, Norway

**Keywords:** adult neurogenesis, fear conditioning, hippocampus, spatial memory

## Abstract

The term “memory strength” generally refers to how well one remembers something. But more precisely it contains multiple modalities, such as how easily, how accurately, how confidently and how vividly we remember it. In human, these modalities of memory strength are dissociable. In this study, we asked whether we can isolate a behavioral component that is dissociable from others in hippocampus-dependent memory tasks in mice, which potentially reflect a modality of memory strength. Using a virus-mediated inducible method, we ablated immature neurons in the dentate gyrus in mice after we trained the mice with hippocampus-dependent memory tasks normally. In memory retrieval tests, these ablated mice initially showed intact performance. However, the ablated mice ceased learned behavior prematurely within a trial compared with control mice. In addition, the ablated mice showed shorter duration of individual episodes of learned behavior. Both affected behavioral measurements point to persistence of learned behavior. Thus, the effect of the postlearning manipulation showed dissociation between initial performance and persistence of learned behavior. These two behavioral components are likely to reflect different brain functions and be mediated by separate mechanisms, which might represent different modalities of memory strength. These simple dissociable measurements in widely used behavioral paradigms would be useful to understand detailed mechanisms underlying the expression of learned behavior and potentially different modalities of memory strength in mice. We also discuss a potential role that immature neurons in the dentate gyrus may play in persistence of learned behavior.

## Significance Statement

We use the term “memory strength” both in everyday life and research settings. Memory strength generally means how well we remember something. However, depending on context, its meaning is varied; it can mean how easily, accurately, vividly and/or confidently we remember it. These different modalities of memory strength are generally correlated but is known to be partly independent. In this study, we asked whether such independent or dissociable behavioral components exist in hippocampus-dependent memory tasks in mice. We provide evidence that initial performance and persistence of learned behavior during memory retrieval tests are dissociable in three memory tasks. Such dissociable components would be helpful to study different modalities of memory strength and their underlying mechanisms in mice in future studies.

## Introduction

The term “memory strength,” which generally means how well one remembers something, is used in both everyday life and research settings. However, its exact meaning can be different depending on situations; you may mean how easily you can remember something, how vividly you remember it, how accurately you remember it, how confidently you remember it or all of them (Bjork and Bjork, 1992; Wozniak et al., 1995; Qin et al., 2011). Although these different modalities of memory strength are often correlated, they are at least partially independent (Squire et al., 2007; Qin et al., 2011; Lacy and Stark, 2013). For example, some of our memories are easy to remember but lack details. Other memories might be difficult to remember but, once remembered, are highly vivid and accurate. It is also possible to have high degree of confidence in a memory that turns out to be false. In human studies, these different modalities of memory strength can be dissociated and have been separately studied (Squire et al., 2007; Geib et al., 2017; Sekeres et al., 2018).

With animal experiments, can we study such different aspects of memory strength? Although the lack of methods to study subjective experience limits experimental approaches for some of the modalities, it should be still possible for other modalities by using objective measurements of behavior during memory tasks. In most rodent studies, we examine memory strength by quantifying the amount or strength of learned behavior, which includes how quickly, how long, how often, how persistently, and how correctly the subject shows learned behavior (Morris et al., 1982; Crawley, 2000; Maren, 2001). However, it is rare to consider different aspects of memory strength. This would be partly because most of commonly used parameters in memory tasks are well correlated with each other (Matzel et al., 2003; Maei et al., 2009), and it is not clear whether any of these behavioral measurements represent a specific modality of memory strength and can be dissociable from others. For example, can how fast/accurate mice start learned behavior be dissociated from how persistently they continue it? In this study, we provide positive evidence for this question. We used a virus-mediated method in mice and induced ablation targeting immature neurons in the dentate gyrus immediately after the mice normally learned hippocampus-dependent memory tasks. We found dissociated effects between simple behavioral measurements reflecting initial performance and persistence of learned behavior.

Original motivation of this study, which is different from the main focus of this report, was to investigate a postlearning role of immature neurons in the adult dentate gyrus. Neurogenesis, the birth of new neurons, persists throughout life in a few regions of the mammalian brain, including the dentate gyrus (Gage, 2000). It has been suggested that newly generated neurons in the adult dentate gyrus play a role in hippocampus-dependent forms of memory (Shors et al., 2001; Snyder et al., 2005; Deng et al., 2009). These new neurons go through a maturational period lasting over a month, during which they exhibit enhanced functional and morphologic plasticity (Wang et al., 2000; Espósito et al., 2005; Zhao et al., 2006; Ge et al., 2007; Tronel et al., 2010; Åmellem et al., 2017). The nature of enhanced plasticity varies between early and late maturational stages (approximately one to three and approximately four to six weeks, respectively) and has been postulated to create multiple time windows during which the neurons can make unique contributions to memory processes (Aasebø et al., 2011). While new neurons in the late maturational stages have been shown to be involved both during and after memory formation (Denny et al., 2012; Gu et al., 2012), those in the early maturational stages have been shown to contribute to memory formation (Deng et al., 2009; Vukovic et al., 2013; Seo et al., 2015). However, a postlearning role of new neurons at the early maturational stage has not been identified.

## Materials and Methods

### Lentiviral vector construction

**Figure 1.**
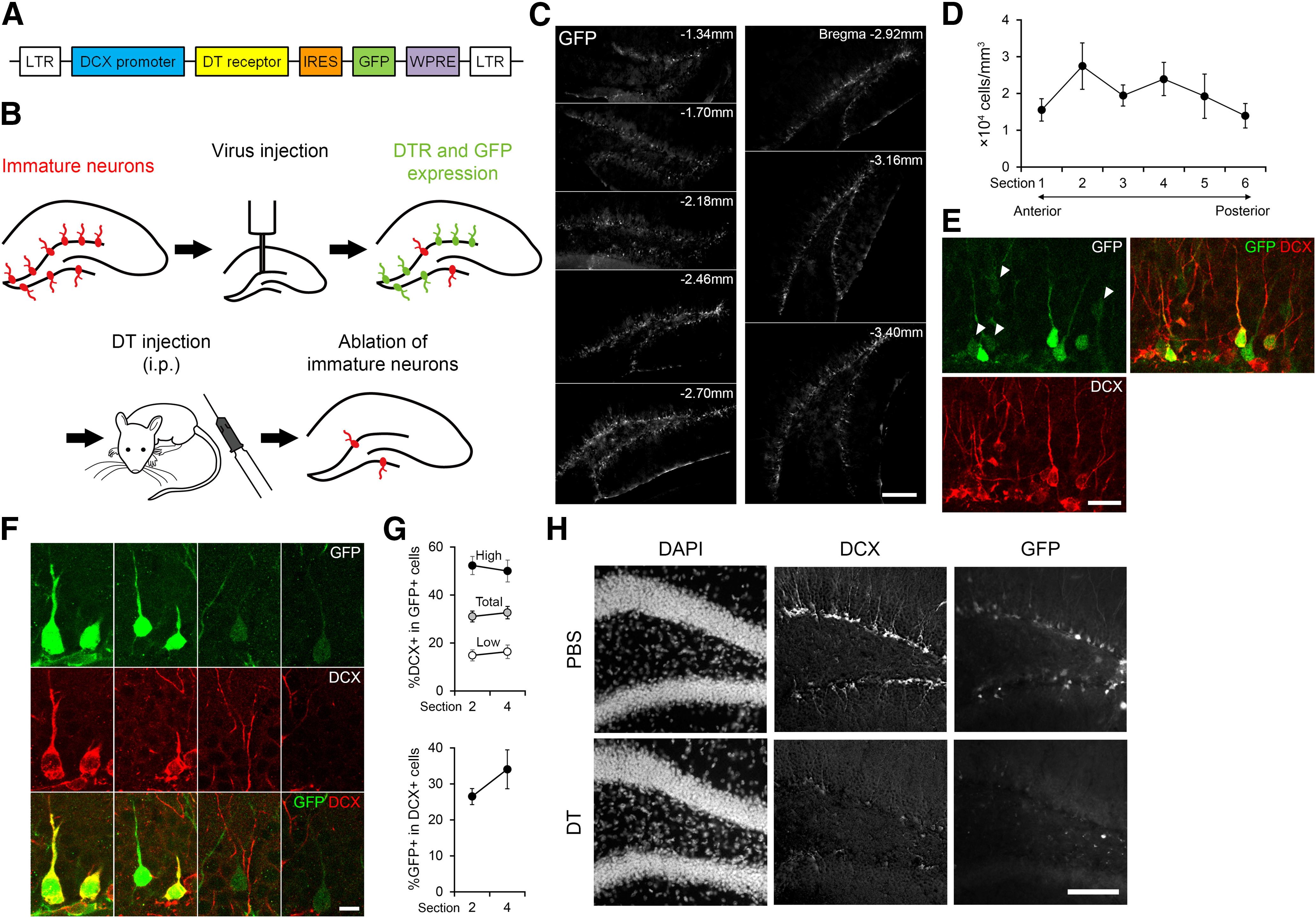
Diphtheria toxin-induced ablation of DCX+ cells in the dentate gyrus using lentiviral transduction of DTR. ***A***, Schematic representation of the recombinant lentiviral vector construct. LTR: long terminal repeat; DCX: doublecortin; DT: diphtheria toxin; IRES: internal ribosome entry site; GFP: green fluorescent protein; WPRE: Woodchuck hepatitis virus posttranscription regulatory element. ***B***, Diagram of the ablation technique specific for immature neurons. ***C–E***, To evaluate the distribution and specificity of viral transduction, we injected the viral vector into the dentate gyrus of mice and examined GFP expression 7 d after the injection, which corresponds to the time when DT was injected in behavioral experiments (Figs. 6-8). ***C***, Representative images showing the distribution of virus-transduced cells visualized by GFP fluorescence over the AP axis of the dentate gyrus. GFP expression was localized predominantly along the hilar border of the granule cell layer across the AP axis of the dentate gyrus, where adult-born neurons are known to be located. Scale bar: 200 μm. ***D***, Density of GFP+ cells across the AP axis of the dentate gyrus. GFP+ cells were quantified in every 12 coronal sections covering the AP axis of dentate gyrus. Sections 1 and 6 correspond to ∼1.06–1.34 and 3.52–3.80 mm posterior to the bregma, respectively. ***E***, A representative confocal image of the granule cell layer in a section immunostained against GFP and DCX. Among 460 DCX+ cells examined (from one hemisphere in three sections each of two mice), 40.7% of DCX+ cells expressed GFP, indicating that viral transduction was achieved in a large proportion of immature neurons. Among 455 GFP+ cells examined (from one hemisphere in three sections each of two mice), 43.8% of GFP+ cells were DCX+. We noted that GFP expression level was highly variable between GFP+ cells (low GFP-expressing cells are highlighted by arrowheads). Scale bars: 20 μm. ***F***, ***G***, To further evaluate the distribution and specificity of viral transduction, we analyzed sections from nine control mice used for the behavioral experiment described in Figure 6. These sections were from 17 d after virus injection, during which water maze training, probe trials and re-training were performed. ***F***, Representative confocal images showing colocalization of DCX and GFP in neurons in the granule cell layer. Scale bars: 10 μm. ***G***, Top, Proportion of GFP+ cells expressing DCX. Percentage within total population, high and low GFP-expressing cell populations were plotted separately. Bottom, Proportion of DCX+ cells expressing GFP. For each population, two sections from different AP levels corresponding to sections 2 and 4 in ***D*** were analyzed separately. We observed some GFP+DCX– cells which appeared to include non-neuronal and granule cells based on their morphology. Without an active degradation mechanism, GFP protein is highly stable even after the transcription activity of the promoter is shut off (Andersen et al., 1998). Therefore, GFP protein is expected to be maintained in new neurons that expressed DCX at the time of virus injection but thereafter lost the expression of DCX (and presumably DTR) during the survival time. Thus, some of GFP+DCX– cells were likely to be new neurons that had just lost DCX expression. In accordance with this possibility, the proportion expressing DCX was higher in high GFP-expressing cells (>50%) than that of low-expressing cells (<20%). Nonetheless, we cannot completely exclude the possibility that some of GFP+ cells were mature neurons. Our estimation revealed that the proportion of GFP+/DCX– cells among the total granule cell population in the dentate gyrus would be small (0.6%; see Materials and Methods). This is consistent with our observation of no ablation of mature granule cells (nine weeks old), shown in Figure 2. ***H***, Fluorescent images of the virus-injected dentate gyrus after the injection of PBS or DT. Top, The granule cell layer visualized by DAPI staining was intact after DT-induced ablation. Middle and bottom, DT injection led to an obvious reduction in DCX+ and GFP+ cells. Scale bar: 100 μm.

A lentiviral transfer vector was designed to express diphtheria toxin (DT) receptor (DTR; Dorland et al., 1979) under the control of the human doublecortin (DCX) promoter (Fig. 1*A*). cDNA encoding human DTR (pcDNA3 proHB-EGF WT, Addgene) was inserted into a lentiviral transfer plasmid under the control of the human DCX promoter (Karl et al., 2005). DTR is the human homolog of heparin-binding EGF-like growth factor in the cell membrane. DTR has a high affinity for DT, which is a protein synthesis inhibitor produced by Corynebacterium diphtheria, and is the cause of DT sensitivity in human cells (Collier, 1975; Iwamoto et al., 1994; Buch et al., 2005). Because the murine homolog of DTR has a low affinity for DT, murine cells are insensitive to the dose used in this study (50 ng/g body weight). Upon expression of DTR in immature neurons using the lentiviral vector, systemic injection of the dose of DT into mice induces the death of immature neurons (Fig. 1*B*). To visualize the transduced cells, cDNA encoding enhanced green fluorescent protein (GFP) was inserted 3′ to the DTR gene following an internal ribosome entry site sequence.

Lentiviral particles were produced using a protocol modified from a previously described method (Tashiro et al., 2015b). After the centrifugation steps described in Tashiro et al. (2015b), the suspension was purified using Lenti-X Concentrator purification columns (Clontech). Amicon Ultra-4 Centrifugal Filter Units (EMD Millipore) were used to perform buffer exchange to sterile Dulbecco’s PBS (DPBS; catalog #14040-174, Life Technologies) and to concentrate the solution to a final volume of 100–150 μl. Aliquots of the lentiviral solution were stored at −80°C until use.

### Subjects

We used male and female C57BL/6 mice bred in our local facility in Norway or Singapore or purchased from Charles River or InVivos. For water maze and fear conditioning tasks, we used female C57BL/6 mice purchased from the two sources above to avoid behavioral variability between two sexes. The mice were 7–12 weeks old at the start of the experiments and were housed in acrylic cages with access to food and water *ad libitum* under a 12/12 h light/dark cycle. All experiments were approved by the Norwegian Animal Research Authority and/or Institutional animal care and use committee at Nanyang Technological University.

### Surgical procedure

The solution containing the lentiviral vector was stereotaxically injected into the dentate gyrus (Tashiro et al., 2015b). The mice were anesthetized with 5% isoflurane [2-chloro-2-(difluoromethoxy)−1,1,1-trifluoroethane] in air at a flow rate of 1000 ml/min. The concentration of isoflurane was gradually reduced to 0.75% or higher, at which concentration deep anesthesia was maintained. The mice were subcutaneously injected with an analgesic (0.15 g/kg Temgesic). For the experiment described in Figure 3 and Figures 5*B–G*, 7, 8, a local anesthetic, Marcaine (AstraZeneca) and lignocaine (1.33 mg/ml, 0.1 ml), respectively, was additionally injected subcutaneously above the skull before performing the skin incision. For the experiment described in Figures 5*B–G*, 7, 8, buprenorphine (0.2 ml, 0.3 mg/ml) was additionally given intraperitoneally before surgery and meloxicam and baytril were additionally given in drinking water (8 and 85 mg/l, respectively) for 3 d after the surgery.

**Figure 2.**
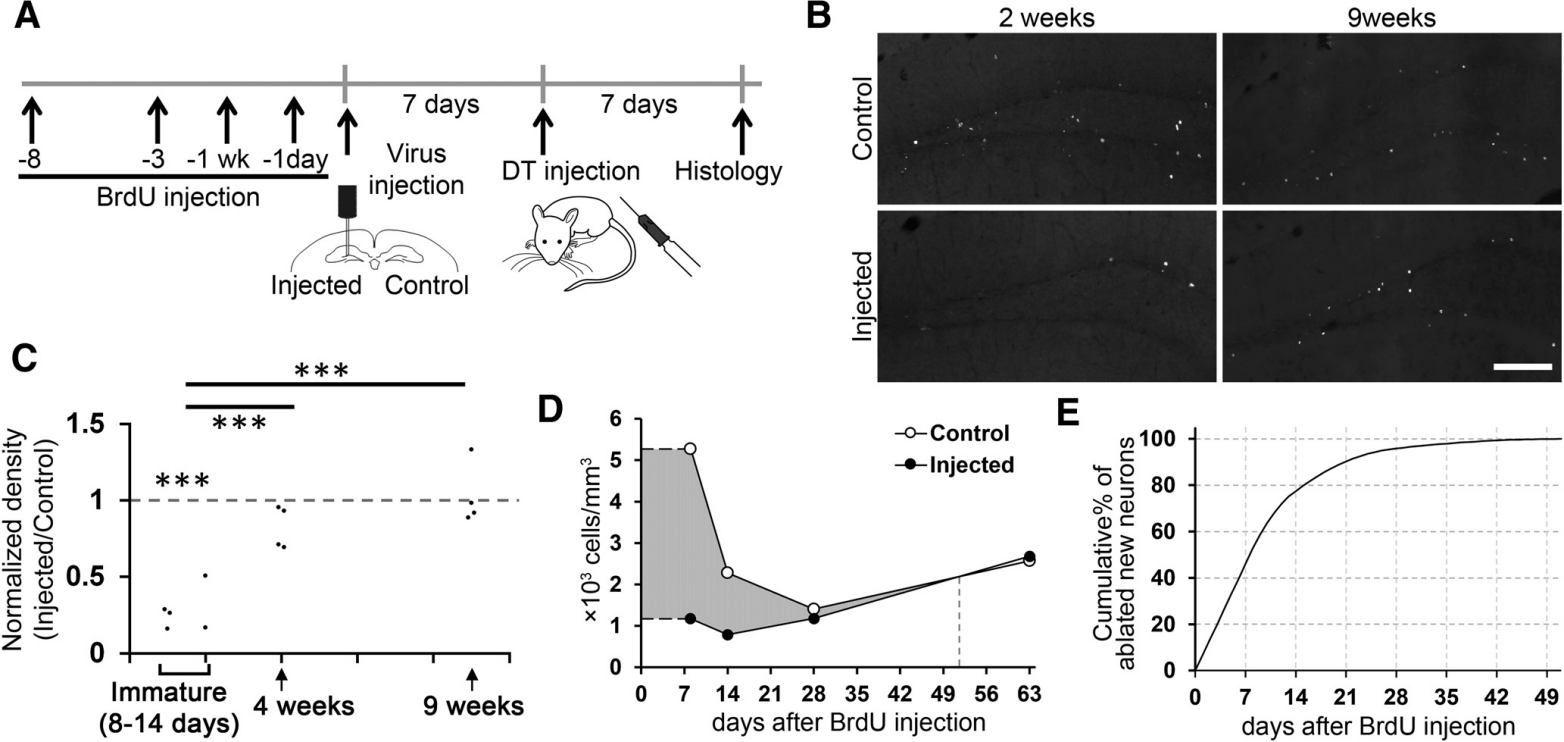
DT-induced ablation of new neurons at the early maturational stage. ***A***, Experimental design. To label the different ages of newborn neurons, BrdU was injected into mice at four different time points (8 d, 14 d, 4 weeks, and 9 weeks) before DT administration. For each mouse, one dose of BrdU (100 μg/g body weight) was injected intraperitoneally. ***B***, Representative images showing two- or nine-week-old BrdU+ cells from virus-injected and non-injected control hemispheres after DT injection. Scale bar: 150 μm. ***C***, Normalized densities of BrdU+/NeuN+ cells. The densities in the injected hemispheres were normalized by dividing by the densities in the non-injected hemispheres of the same mice. Each data point represents one mouse (8 d: *n* = 3 mice, two weeks: *n* = 2, four weeks: *n* = 4, nine weeks: *n* = 4). A value of 1 indicates that the densities of both hemispheres are equal. Note that a significant reduction in immature (8- to 14-d-old) neurons but minimal effects on four- and nine-week-old neurons. The densities were measured from three sections (every 12 40-μm-thick sections, corresponding to sections 2–4 in Fig. 1*D*) for each mouse. These three sections are distributed over the dorsal region of the dentate gyrus. ***D***, Estimation of the numbers of ablated neurons at different ages, using trapezoidal rule integration. Difference in the number of new neurons between the control and injected hemisphere (gray area) reflects the number of ablated new neurons. ***E***, Cumulative percentage of ablated new neurons at different ages, using trapezoidal rule integration. Percentages of ablated neurons younger than different ages in the total ablated population were plotted; ***p < 0.005.

**Figure 3.**
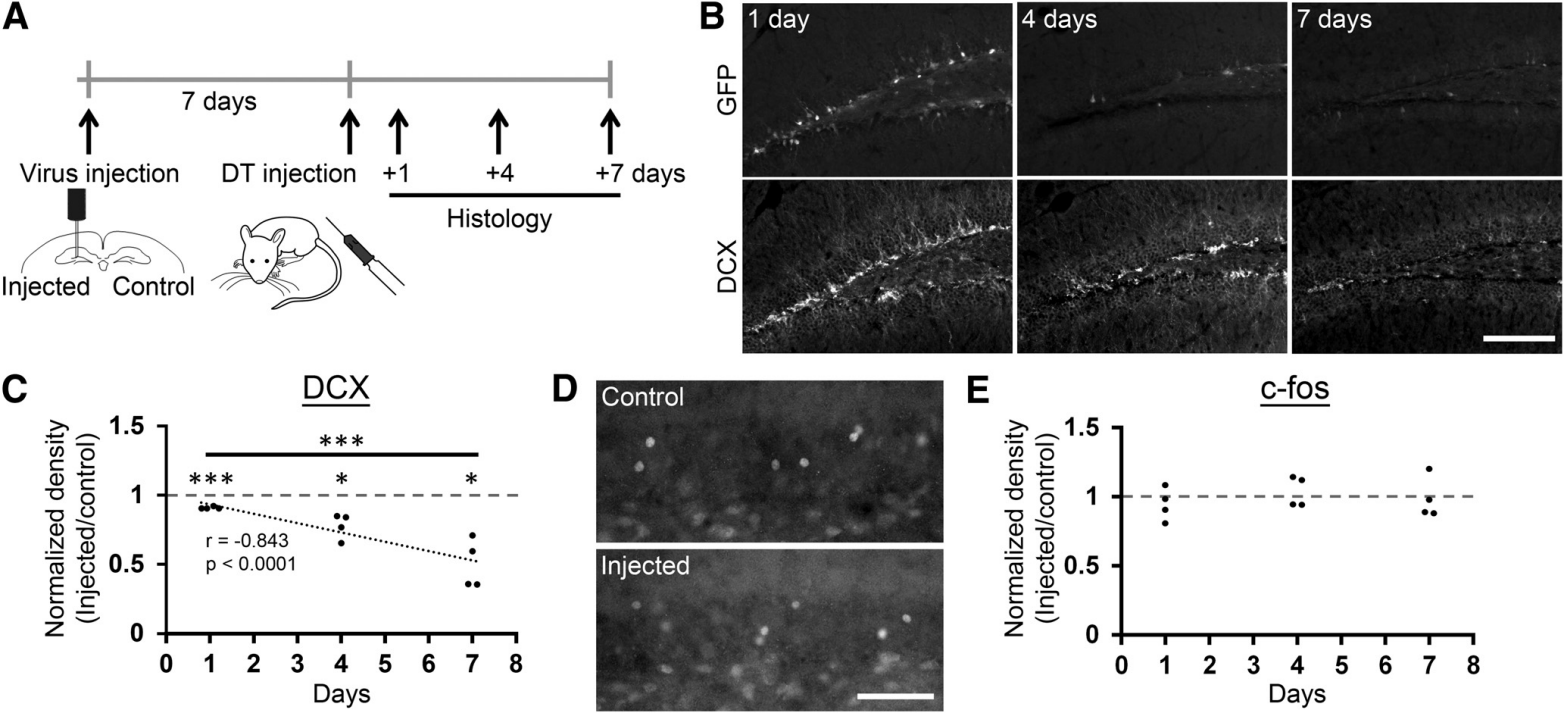
Ablation of DCX+ cells occurs over multiple days while keeping the ability of protein synthesis/activity-dependent gene expression intact in the granule cell layer. ***A***, Experimental design. ***B***, Representative images showing the reduction of GFP+ cells over 7 d after DT injection. Scale bars: 150 μm. ***C***, Normalized densities of DCX+ cells in the virus-injected hemisphere after DT injection. The densities in the injected hemispheres were normalized by dividing by the densities in non-injected hemispheres in the same mice. ***D***, Representative images of c-fos expression in the granule cell layer of virus-injected and non-injected control hemispheres. Scale bars: 100 μm. ***E***, Normalized densities of c-fos+ cells over 7 d after DT injection. The same way of normalization as ***C*** was used.

**Figure 4.**
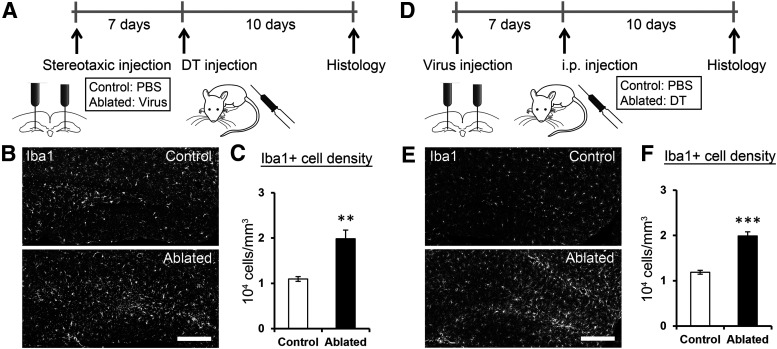
Ablation method induces Iba1 expression in the dentate gyrus. ***A***, Experimental design. Mice were stereotaxically injected with PBS (control group) or the viral vector (ablated group) into the dentate gyrus. After 7 d of recovery, DT was injected into both groups. Brain sections were prepared from the mice after an additional 10 d of survival. ***B***, Representative images of Iba1 immunostaining of brain sections from the control and ablated groups. ***C***, Density of Iba1+ cells in the dentate gyrus. An increase was observed after the induction of ablation by a combination of the viral vector and DT injections (*p* = 0.0081, *t*_(4.566)_ = −4.478, *n* = 5 for each group, independent sample *t* test). ***D***, Experimental design. Mice were stereotaxically injected with the viral vector into the dentate gyrus. After 7 d of recovery, PBS (control group) or DT (ablated group) was injected into both groups. Brain sections were prepared from the mice after an additional 10 d of survival. ***E***, Representative images of Iba1 immunostaining of brain sections from the control and ablated groups. ***F***, Density of Iba1+ cells in the dentate gyrus. An increase was observed after the induction of ablation by a combination of the viral vector and DT injections (*p* = 3.9 × 10^−5^, *t*_(8)_ = −8.132, *n* = 5 for each group, independent sample *t* test).

**Figure 5.**
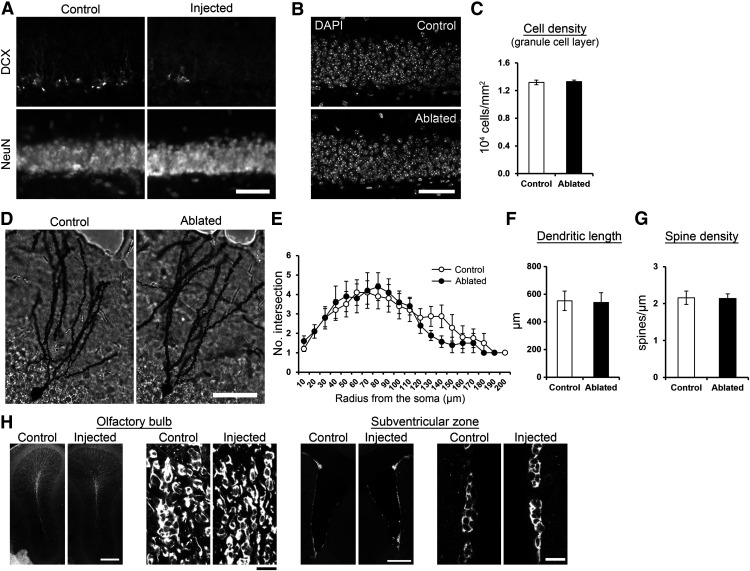
Overall integrity of the granule cell layer and mature granule cells is intact after DT-induced ablation. ***A***, NeuN immunostaining showed the integrity of granule cell layer after DT-induced ablation. The lentiviral vector was injected into the dentate gyrus in one hemisphere. Seven days later, DT was injected. After another 7-d survival, brain sections were prepared and immunostained with DCX or NeuN. The images were from virus-injected (Injected) and non-injected (control) hemispheres. Scale bar: 50 μm. ***B***, DAPI immunostaining showing the integrity of granule cell layer of mice in the control and ablated group. Scale bar: 50 μm. ***C***, Cell density in the granule cell layer was not affected by the ablation (*p* = 0.783, *t*_(23)_ = −0.279, control: *n* = 13 mice, ablated: *n* = 12, independent sample *t* test). The analysis was performed using the sections from the mice used in the experiments described in Figure 7. ***D***, Golgi-stained granule cells of mice in the control and ablated group. The lentiviral vector was injected into the dentate gyrus in both hemisphere, and PBS or DT was systemically injected 7 d later. After another 7-d survival, the mice were euthanized for the Golgi staining. Scale bar: 50 μm. ***E***, Sholl analysis for Golgi-stained granule cells in the control and ablated group. No significant group difference was detected (group: *p* = 0.600, *F*_(1,271)_ = 0.276, radius: *p* = 1.1 × 10^−9^, *F*_(19,271)_ = 4.429, group × radius interaction: *p* = 0.996, *F*_(18,271)_ = 0.330, *n* = 10 cells for each group, two-way ANOVA). ***F***, Total dendritic length of the Golgi-stained granule cells. No significant, group difference was detected (*p* = 0.907, *t*_(18)_ = 0.119, *n* = 10 cells for each group, independent sample *t* test). ***G***, The density of dendritic spines in the Golgi-stained granule cells. No significant group difference was detected (*p* = 0.923, *t*_(18)_ = 0.098, *n* = 10 cells for each group, independent sample *t* test). ***H***, Intact olfactory bulb neurogenesis after DT-induced ablation in the dentate gyrus. Fluorescent images of DCX+ cells in the olfactory bulb and subventricular zone after DT-induced ablation in the dentate gyrus. Images from virus-injected and control hemispheres are shown. To confirm that the ablation technique did not affect neurogenesis outside the dentate gyrus, we injected the viral vector into the dentate gyrus in one hemisphere and performed DT injection one week after the virus injection. We examined the densities of DCX+ cells one week after the DT injection and did not observe any obvious difference between the virus-injected hemisphere and non-injected control hemisphere either in the olfactory bulb or in the subventricular zone. This observation was confirmed by quantifying the density of DCX+ cells in the subventricular zone (control hemisphere: 2.97 ± 0.02; ablated hemisphere: 3.22 ± 0.15 in 10^3^ cells/mm^2^, *p* = 0.248, *t*_(2)_ = −1.614, *n* = 3 mice, paired *t* test) and the granule cell layer of the olfactory bulb (control hemisphere: 5.35 ± 1.47; ablated hemisphere: 5.68 ± 1.14 in 10^4^ cells/mm^3^, *p* = 0.825, *t*_(2)_ = −0.252, *n* = 3 mice, paired *t* test). Scale bars: 300 μm (left) and 15 μm (right); **p < 0.01, ***p < 0.005. Scale bars: 300 μm.

**Figure 6.**
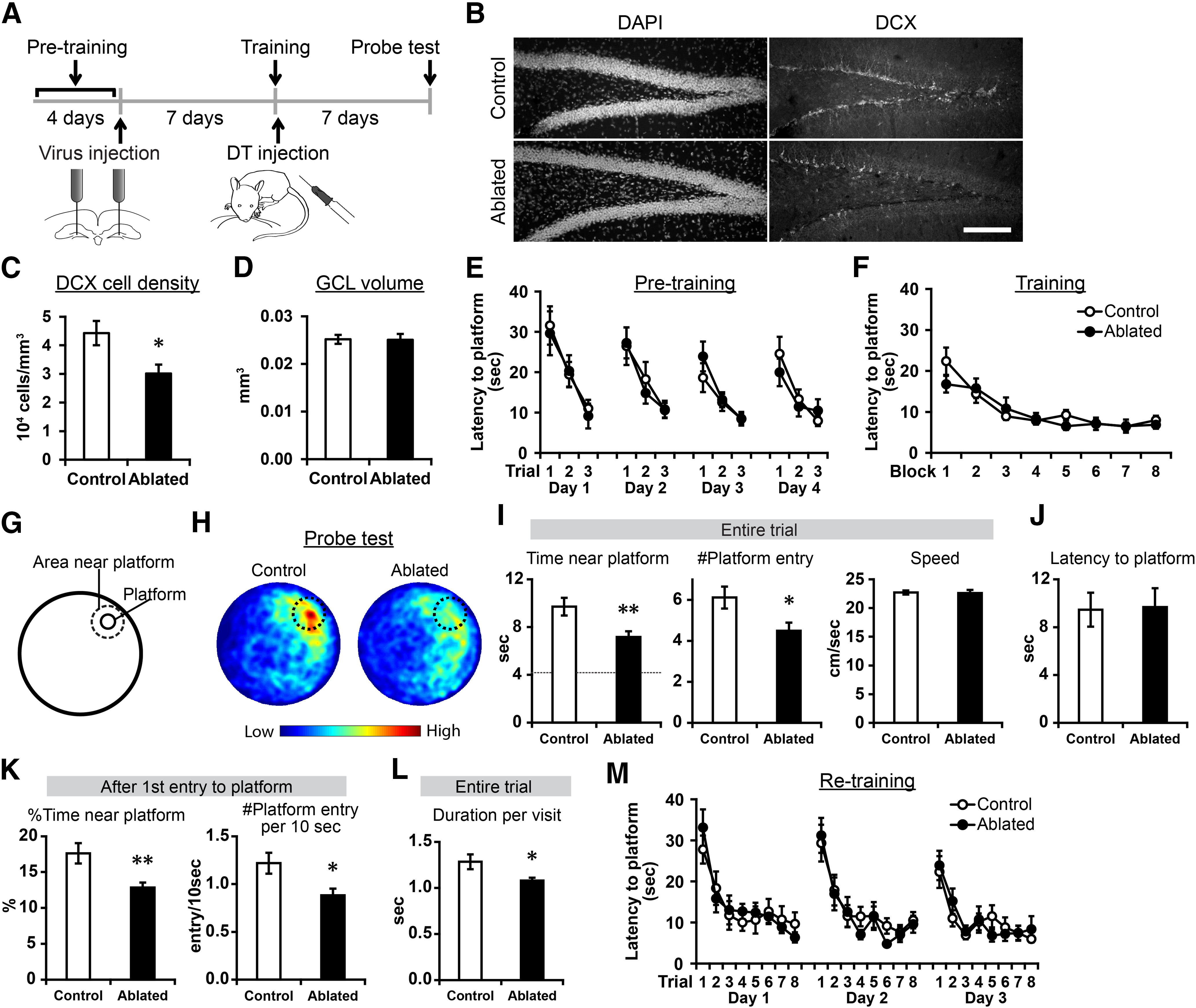
Posttraining ablation impaired the persistence of platform search behavior in a water maze task. ***A***, Experimental design. ***B***, Representative images of DAPI staining and DCX+ cells in the dentate gyrus of mice in the ablated and control groups. Scale bar: 150 μm. ***C***, The densities of DCX+ cells. ***D*,** The volume of the granule cell layer. ***E***, ***F***, Latency to locate the platform during pretraining (***E***) and training (***F***). The two groups improved performance similarly well in both pretraining and training. ***G***, The position of the removed platform and the area near the platform in probe trials. The area near the platform is defined as the circular area within 14 cm of the center of the former platform position. ***H***, Occupancy plots showing average time that mice spent at different positions in the pool. The color bar below shows the color code for occupancy time; warmer colors indicate high occupancy, while cooler colors represent low occupancy. Dotted circles indicate the area near the platform. ***I***, Time spent in the area near the platform position, the number of platform entries and swimming speed in probe tests. A dotted line indicates the chance level in time spent near the platform position. ***J***, Latency to reach the position of the removed platform. ***K***, Percentage of time spent in the area near the platform position and the number of platform entries per 10 s after the first entries into the position of the removed platform. ***L***, Duration per visit to the area near the platform. ***M***, Latency to locate the platform during re-training. The two groups improved performance similarly well; *p < 0.05, **p < 0.01.

**Figure 7.**
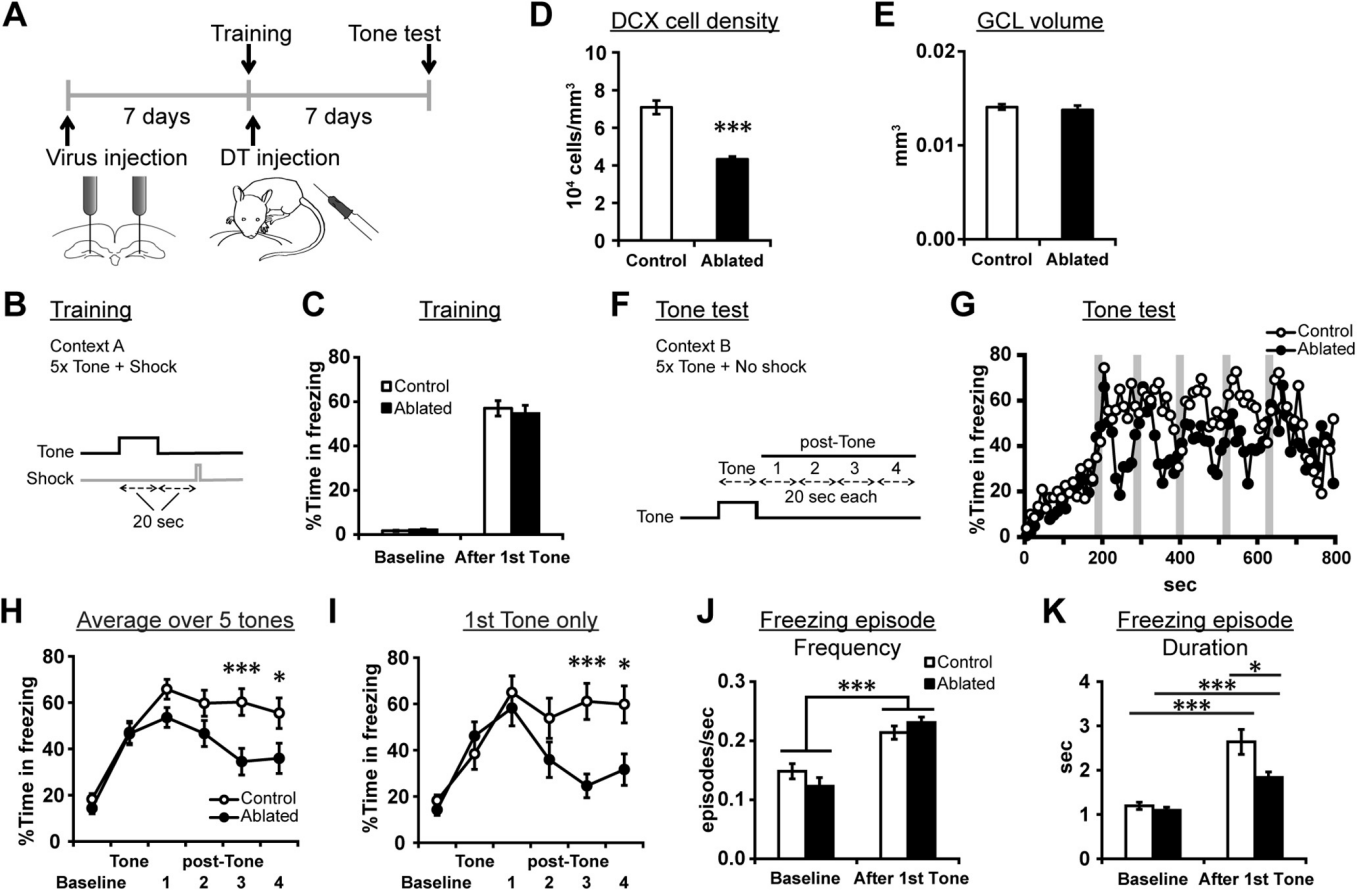
Posttraining ablation impaired the persistence of tone-induced freezing in a tone trace fear conditioning task. ***A***, Experimental design. ***B***, Timings of a tone (conditional stimulus) and an electrical shock (unconditioned stimulus) during training. ***C***, Percentage time in freezing during the baseline period and after the onset of first tone during training. No significant difference was detected between the groups. ***D***, The densities of DCX+ cells. ***E***, The volume of the granule cell layer. ***F***, The definition of the tone and posttone periods in the tone test. ***G***, Percentage time in freezing for every 10 s during the tone test. Gray areas indicate the timing of tone deliveries. ***H***, ***I***, Percentage time in freezing during baseline, tone, posttone 1, 2, 3, and 4 periods, averaged over five tones (***H***) or for first tone only (***I***). ***J***, ***K***, Frequency (***J***) and duration (***K***) of freezing episodes during the baseline period and after the onset of first tone in the tone test; *p < 0.05, ***p < 0.005.

**Figure 8.**
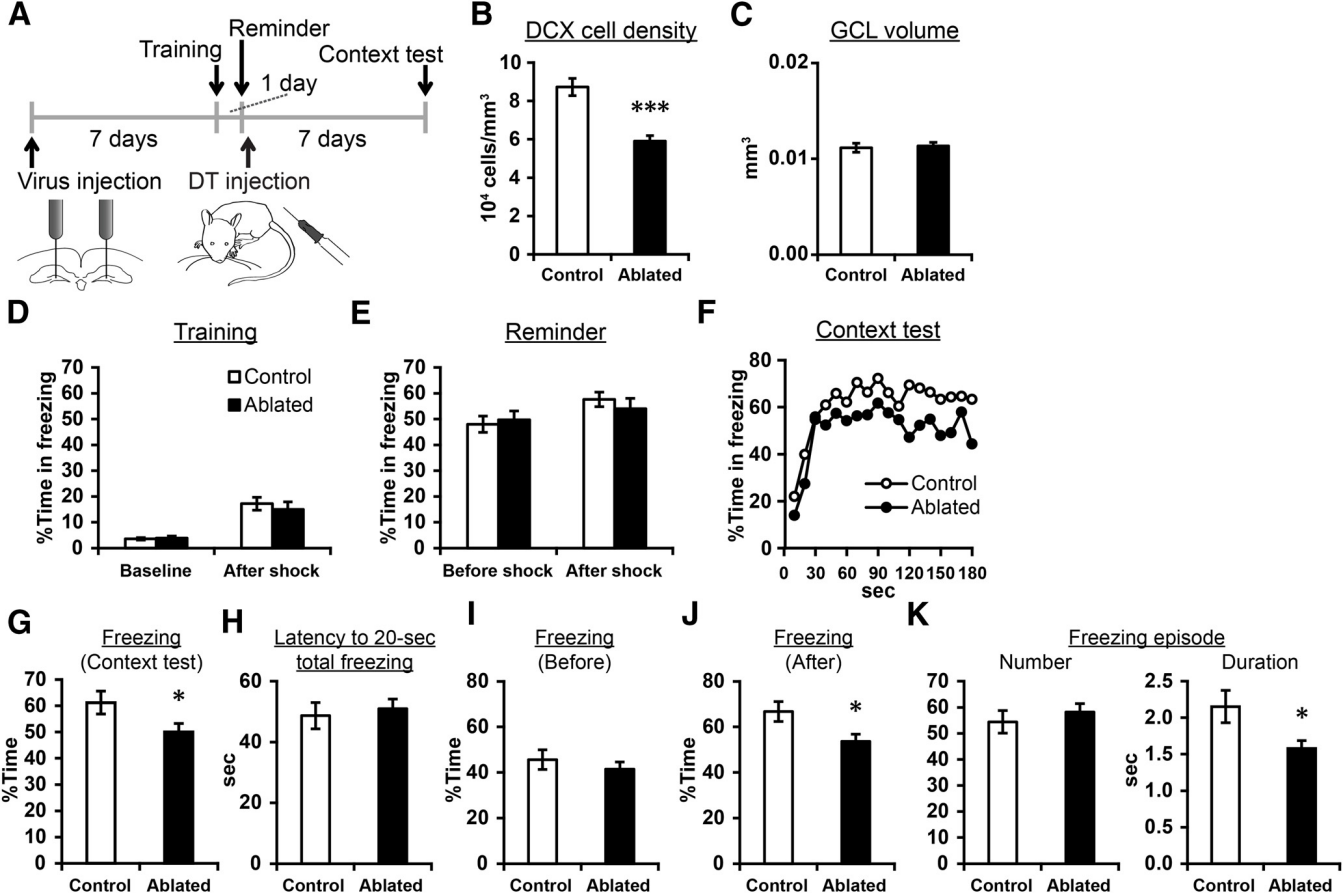
Posttraining ablation impaired the persistence of context-induced freezing in a contextual fear conditioning task. ***A***, Experimental design. ***B***, The densities of DCX+ cells. ***C*,** The volume of the granule cell layer. ***D***, ***E***, Percentage time in freezing during the baseline period and after the shock in training (***D***) and remainder training (***E***). No significance different was detected between the groups. ***F***, Percentage time in freezing for every 10 s during the context test. ***G***, Percentage time in freezing in the context test. ***H***, Latency to reach 20-s cumulative freezing in the context test. ***I***, ***J***, Percentage time in freezing before (***I***) or after (***J***) reaching cumulative freezing of 20 s. ***K***, The number and duration of freezing episodes; *p < 0.05, ***p < 0.005.

The lentiviral solution was drawn into a microsyringe equipped with a blunt-end 33-gauge needle (Hamilton Company). After the skull was exposed, one or two holes were drilled at the anteroposterior (AP) coordinate: −1.8 mm; medial-lateral (ML): +/−1.8 mm from the bregma except for the experiment described in Figures 5*B–G*, 7, 8, where the coordinate of AP: −2.0 mm, ML: +/−1.3 mm was used. The tip of the needle was inserted into the brain at the same AP and ML coordinates and lowered to 2.3 mm ventrally from the skull surface. In each injection, 1.5 μl of lentiviral solution was infused at a rate of 2.5 nl/s (or 3.33 nl/s for the experiment described in Fig. 5*B–G*, 7, 8). The titer of the lentiviral solution was 0.1–0.5 × 10^5^ GFP+ colony-forming units/ml in HEK 293FT cells (Life Technologies). In the experiments described in Figures 1-3, 5*A*,*H*, the viral vector was injected into one hemisphere. For Figures 2, 3, 5*A*,*H*, the other, non-injected hemisphere was used as a control. In the experiments described in Figure 1*H*, control mice were injected with PBS. In the experiments described in Figures 4, 5*B–G*, 6–8, the vector or vehicle was injected into both hemispheres.

### DT injection

DT (Calbiochem) was dissolved in DPBS at a final concentration of 5 μg/ml. The mice received a single dose of DT (50 ng/g body weight) intraperitoneally 7 d after viral injection.

### 5-Bromo-2’-deoxyuridine (BrdU) injection

We dissolved BrdU (catalog #B5002, Sigma) in 0.9% saline at a concentration of 10 mg/ml, and the solution was filter sterilized. For each mouse, one dose of BrdU (100 μg/g body weight) was intraperitoneally injected.

### Novel environment exploration

To induce the activation of granule cells in the dentate gyrus, we placed the mice in a novel environment (an open field consisting of a 32 × 28 × 28 cm plastic box with black-striped and white-striped walls). The mice were allowed to explore the novel environment for 15 min. The mice underwent perfusion fixation 90 min after the novel environment exposure.

### Histology

The preparation of fixed brain sections has been previously described (Tashiro et al., 2015a). Forty-micrometer-thick coronal sections were collected from the AP level covering the entire hippocampus (∼1.22 to 3.80 mm posterior to the bregma). The sections were stored in a cryoprotectant solution (20% glycerin, 30% ethylene glycol in 0.1 m PB) at −20°C until use. After being rinsed with 0.1 m Tris-buffered saline (TBS), the sections were incubated with a blocking solution [0.25% Triton X-100 (Sigma) and 3% donkey serum (Sigma) in 0.1 m TBS] for 60 min to minimize non-specific immunoreactions. The sections were incubated with primary antibodies in the blocking solution for 2 d at 4°C. Unbound primary antibodies were removed by washing with 0.1 m TBS, and the sections were then incubated with secondary antibodies in the blocking solution for 4 h at room temperature. Nuclear staining with 4’,6-diamidino-2-phenylindole-dihydrochloride (DAPI; MERCK) was performed. The primary antibodies used were rabbit anti-c-fos (1:500; Santa Cruz Biotechnology catalog #sc-52), goat anti-DCX (1:500 or 1:400; Santa Cruz Biotechnology catalog #sc-8066), rat anti-GFP (1:500; Nacalai Tesque catalog #04404-84), rabbit anti-GFP (1:500; Life Technologies catalog #A11122), mouse anti-NeuN (1:500; Millipore catalog #MAB377), rat anti-BrdU (1:1000; AbD Serotec catalog #OBT0030G), and goat anti-Iba1 (1:500; Abcam catalog #ab5076). All secondary antibodies were purchased from Jackson ImmunoResearch Laboratories and used at 1:600 or 1:250 dilutions. The secondary antibodies used were donkey anti-rabbit-DyLight 488 (catalog #711-485-152), donkey anti-rat-Alexa 488 (catalog #712-545-153), donkey anti-rat-Cy3 (catalog #712-165-153), donkey anti-goat-DyLight 649 (catalog #705-495-147), donkey anti-mouse-DyLight 649 (catalog #715-495-151), and donkey anti-goat DyLight 549 (catalog #705-505-147). Golgi staining was performed using FD Rapid GolgiStainTM kit (catalog #PK401, FD NeuroTechnologies).

### Image analysis

Epifluorescence imaging and cell counting were performed with Axio Scope A1 or Axio Imager M1 microscopes (Zeiss) with 5×, 10×, and 20× objective lenses. Confocal imaging was conducted using an LSM710 confocal microscope (Zeiss) equipped with 488-, 543-, and 633-nm laser lines and ZEN image-acquisition software. ImageJ software (National Institutes of Health) was used to count cells in confocal images and measure the area of granule cell layers defined by DAPI staining. The volume of the granule cell layer for individual sections was calculated by multiplying the area of the granule cell layer by the thickness of the section (40 μm). Cell density was calculated by dividing the cell number by the volume of the granule cell layer. The normalized cell density indicated in Figures 2, 3 was calculated as the density in the ablated hemisphere divided by the density in the contralateral non-injected hemisphere of each mouse.

The proportion of GFP+/DCX– cells in the total granule cell population was estimated using previously reported estimates of granule cell density in the granule cell layer (3.3 × 10^6^ cells/mm^3^; Abusaad et al., 1999). The number of GFP+/DCX– cells and the volume of the granule cell layer were measured from confocal images covering the entire granule cell layer of three sections per mouse. These sections were selected from comparable rostro-caudal levels between mice.

For Figure 2*E*, we estimated the age of ablated new neurons as follows. Difference in the number of new neurons from the control and injected hemisphere reflects the number of ablated new neurons. We estimated the number of ablated new neurons at different ages using the following three extrapolations. (1) We used the value on day 8 to estimate the number on days 1–7. (2) The numbers between examined time points were estimated by the trapezoidal rule. (3) We assumed that new neurons up to 51 d old were ablated. This is because, with the trapezoid rule, the density in the control hemisphere becomes lower than that in the injected hemisphere from day 52 onwards, and the numbers of ablated new neurons become negative. Using these assumptions, the number of ablated neurons was estimated by calculating the area in the graph which is below the line formed by control hemisphere data and above the line formed by the injected hemisphere (Fig. 2*D*, gray area).

Confocal z-stacks for Golgi-stained granule cells were acquired, and the dendrites of Golgi-stained granule cells were traced using Neuromantic software (Myatt et al., 2012). Sholl analysis was performed in ImageJ software with Fiji package (Schindelin et al., 2012). Dendritic spines were counted under Axio Scope A1 microscope with a 100× objective lens (Zeiss).

### Water maze task

Training in a series of water maze tasks was performed in a 1-m diameter pool placed in a dimly lit room. Dark blue curtains displaying two visual cues covered the area around the pool. The pool was filled with 17–21°C water ∼30 cm deep. The water was made opaque with white, non-toxic paint. The mice were moved to the water maze room in Plexiglas cages and allowed to habituate to the room for 5–10 min before the beginning of the experiments. For all trials except the probe trials, the mice were placed in the pool facing the wall and left to swim until they located a transparent Plexiglas circular platform (11 cm in diameter). The mice were removed from the pool after 30 s on the platform for the first trial of the day or 15 s in subsequent trials. When the mice failed to locate the platform within 60 s, they were directed toward the platform by the experimenter using a finger to point at the platform’s position, and the experimenter waited for the mice to climb onto the platform.

“Pretraining” using a visible platform was performed over 4 d before surgery. The platform was made visible to the mice by adjusting the water level 1–2 cm below the platform. At the beginning of each day, the platform was moved to a new position 10 cm from the wall and distributed in four quadrants (NW, NE, SW, and SE). The mice underwent three trials per day. The starting positions were pseudorandomly determined from the three locations at 90°, 180°, and 270° from the platform.

One to 3 d after the completion of pretraining, viral vector injections into both hemispheres were performed. After 7 d of recovery, “training” using a hidden platform was performed in 1 d. The platform was submerged 1 cm below the water surface. The starting positions were pseudorandomly determined from the four locations at 45°, 135°, 225°, and 315° from the platform. Four blocks of three trials were performed in both the morning and the afternoon (a total of 24 trials), with a 6-h interval between the morning and afternoon sessions. The platform position was identical throughout the 24 trials. Between 5 and 10 min from the end of the last training trial, the mice were injected intraperitoneally with either DT (50 μg/kg) or an equal volume of vehicle (DPBS).

After 7 d, spatial memory for the platform position used during training was tested in probe trials. The platform was removed from the pool, and the mice in small, clean Plexiglas cages were moved individually to the water maze room before their trials. After being transported to the water maze room, the mice were left alone for 3 min. The mice were then placed into the pool facing the wall and allowed to swim for 1 min in the pool. Three probe trials were conducted for each mouse with inter-trial intervals of 1 min. To examine memory performance without an effect of memory extinction, data from the first trials were analyzed.

Starting 1 d after the probe trials, the mice underwent “re-training” with eight trials per day for three consecutive days. The platform was submerged 1 cm below the water surface. The platform was moved to a new position each day in one of three quadrants that had not been used during training or previous re-training days. The starting position was pseudo-randomly chosen from four locations at 45°, 135°, 225°, and 315° from the platform. After the final re-training trial, the mice were perfusion fixed with 4% paraformaldehyde in 0.1 m PB.

All trials were recorded with a video camera placed over the pool and analyzed with the ANY-maze software (Stoelting). The first probe test of one control mouse was accidentally not recorded, and data for this mouse were removed from the probe test analysis. The chance level of time spent near the platform was calculated based on the area ratio of near-platform zone to the whole pool. For occupancy plots, the time series of position data extracted from the ANY-maze software were smoothed with a two-dimensional Gaussian filter and used to calculate the time spent in each pixel of the pool using custom-made programs written in MATLAB software (The MathWorks). The platform position was slightly different between groups of animals trained separately. We made the occupancy plots by rotating the entire arena so that the angles of platform centers are always at 45°. Radial distance from the pool center has only a few pixels difference between animals.

### Fear conditioning

Fear conditioning was performed in a conditioning chamber (24 cm wide × 20 cm deep × 30 cm high, Ugo Basile). Shortly after mice were moved into the chamber, the behavior of mice started being recorded with an overhead infrared red light camera. The data were analyzed for freezing behavior using EthoVision software (Noldus), where ≥0.5-s continuous inactivity was detected as a freezing episode. The onset of a freezing episode was recorded as the timing of the freezing episode. After virus injection, mice were handled for 10 min/d for 5 d.

The protocol of trace fear conditioning generally followed Seo et al. (2015) with modifications. Training was conducted in context A, where a floor and walls were made of steel rods and acrylic plates, respectively, the floor and walls were cleaned with 70% ethanol before each trial and were scented with acetic acid. Training was performed in a trace conditioning protocol, which was consisted of five pairings of a tone (5 kHz, 75 dB, 20 s) and an electrical foot-shock (0.4 mA, 2 s) with a 20-s interval. Tone delivery started at 180, 370, 620, 900, and 1060 s after the start of recording. Foot-shock delivery started at 220, 410, 660, 940, and 1100 s after the start of recording. One week after the training, the tone tests were conducted in context B, where the floor was a white plastic plate and the walls were a plastic plate with a stripe pattern and the floor and wall were cleaned with Clorox Fresh Scent before a test for each mouse. Tone delivery started at 180, 280, 390, 510, and 620 s after the start of recording without pairing with a foot-shock. Data from the first tone test were analyzed. Because of accidental cessation of the task, abnormal morphology of the dentate gyrus or failure of DT or virus injections, we removed seven mice. The total numbers of mice included in the analysis were 13 and 12 mice for the control and ablated groups, respectively.

The protocol of contextual fear conditioning generally followed Deng et al. (2009) with modifications. Training, reminder training and contextual tests for contextual fear conditioning were conducted in context A. During training, mice received an electrical foot-shock (0.7 mA, 2 s) 180 s after the start of recording. The mice stayed there for additional 120 s before being removed from the chamber. During reminder training conducted next day, the mice received an electrical foot-shock (0.7 mA, 0.2 s) 180 s after the start of recording. The mice stayed there for additional 120 s before being removed from the chamber. During contextual tests, the mice stayed in the chamber for 180 s after the start of recording. No foot-shock was given. Data from the first contextual test were analyzed. Because of failure of virus injections, we removed two mice. The total numbers of mice included in the analysis were 20 and 18 mice for the control and ablated groups, respectively.

### Experimental design and statistical analyses

Statistical analyses were performed with SPSS software (IBM) and ESCI (https://thenewstatistics.com/itns/esci/). For independent *t* tests (Figs. 4*C*,*F*, 5*C*,*F*,*G*, 6*C*,*D*,*I–L*, 7*C*–*E*, 8*B–E*,*G–K*), we first performed “Levene’s test for equality of variances” in SPSS. When *p* < 0.05, a *t* test without the assumption of unequal variances was performed. When *p* > 0.05, a *t* test with the assumption of equal variances was performed. For two-way ANOVA (Fig. 5*E*), group and radius were used as between-subject and within-subject factors, respectively. For repeated-measures ANOVA (Figs. 2*C*, 3*C*,*E*, 6*E*,*F*,*M*, 7*H–K*), we first performed Mauchly’s test of sphericity. When *p* < 0.05 for a given parameter, the Huynh-Feldt correction was applied. When interaction is significant, preplanned simple effects tests were performed by independent-sample (Figs. 2*C*, 3*C*, 7*H*,*I*,*K*), one sample (Figs. 2*C*, 3*C*), or paired (Fig. 7*K*) *t* tests. Within-subject factors are hemisphere (Fig. 2C, 3C, E), day (Fig. 6*E*,*M*), trial (Fig. 6*E*,*M*), block (Fig. 6*F*), and period (Fig. 7*H–K*). Between-subject factor is maturation stage (Fig. 2*C*), day (Fig. 3*C*,*E*), and group (for all). All *p* values are from two-tailed tests. All data in text and figures are presented as the mean ± SEM. Sample size was determined considering previous studies using similar types of experiments.

### Data availability

The data that support the findings of this study are available from the corresponding author on reasonable request.

## Results

### Characterization of virus-mediated, inducible ablation method

To examine a postlearning role of immature neurons at the early maturational stage defined by DCX expression (Brown et al., 2003), we developed a lentiviral vector bicistronically expressing DTR and GFP under the control of the DCX promoter (Fig. 1*A*,*B*). DCX is expressed in neuronal progenitors and immature neurons in the dentate gyrus (Brown et al., 2003). In adult mice, DCX expression starts declining at approximately two weeks after neuronal birth and then decreases further along neuronal maturation (Kempermann et al., 2003). Therefore, DTR expression is expected to be limited to neuronal progenitors and immature neurons. GFP expression was localized predominantly along the hilar border of the granule cell layer across the AP axis of the dentate gyrus, where adult-born neurons are located (Fig. 1*C*,*D*). Immunostaining for DCX revealed that up to 40.7% of DCX+ cells expressed GFP, indicating that viral transduction was achieved in a large proportion of immature neurons (Fig. 1*E–G*). As a proof of principle for the ablation method, we injected the viral vector into the dentate gyrus, and one week later, we intraperitoneally injected DT for ablation or PBS as a control. After an additional one-week survival period, we perfused the mice and examined DCX+ cells in the dentate gyrus. We observed a clear reduction in the density of DCX+ cells in DT-injected mice compared with that in PBS-injected controls (Fig. 1*H*; quantitative results of larger cohorts in Fig. 6*C*, 7*D*, 8*B*).

Next, to determine the maturational stage of ablated neurons, we labeled different ages of adult-born neurons by injecting a thymidine analog, BrdU, into three groups of mice 8–14 d (for DCX+ immature neurons), four weeks (for young neurons mostly DCX-negative) and nine weeks (for mature neurons) before DT injection (Fig. 2*A*). One week before the DT injection, the viral vector was injected into the dentate gyrus of one hemisphere of all mice. We perfused the mice one week after the DT injection and analyzed the density of adult-born neurons (BrdU+/NeuN+ cells; Fig. 2*B*,*C*). The normalized density and age of adult-born neurons showed a significant, positive correlation (*r* = 0.802, *p* = 9.7 × 10^−4^, *n* = 13 mice), indicating that younger neurons were more selectively eliminated by the ablation method. Further, the normalized densities of 8- to 14-d-old neurons were significantly reduced in the granule cell layer of the virus-injected hemisphere compared with the control hemisphere [65.8 ± 5.7% reduction; two-way (hemisphere × maturation stage) repeated measures ANOVA, hemisphere: *p* = 5.9 × 10^−4^, *F*_(1,10)_ = 24.368, maturation stage: *p* = 2.2 × 10^−4^, *F*_(2,10)_ = 22.059, hemisphere × maturation stage: *p* = 2.2 × 10^−4^, *F*_(2,10)_ = 22.059, *n* = 13 mice in total; *p* = 3.3 × 10^−4^, *t*_(4)_ = −11.440, *n* = 5 mice, one sample *t* tests against 1]. On the other hand, the normalized density of four- and nine-week-old neurons did not show a significant reduction (four weeks: *p* = 0.449, *t*_(3)_ = −0.869; nine weeks: *p* = 0.474, *t*_(3)_ = 0.816, *n* = 4 mice for each, one sample *t* tests against 1). The normalized densities of 8- to 14-d-old neurons were significantly lower than four-week-old neurons (*p* = 0.0028, *t*_(7)_ = −4.502, independent-sample *t* test] and nine-week-old neurons (*p* = 8.7 × 10^−5^, *t*_(7)_ = −8.057). It is known that DCX expression occurs in >80% of new neurons at less than two weeks of neuronal age, reduces to a large extent at four weeks and is then virtually none at neuronal maturity (Jagasia et al., 2009; Snyder et al., 2009). Thus, the pattern of ablation is consistent with DCX expression along neuronal maturation. Furthermore, 77.4 and 90.2% of the ablated new neurons are estimated to be younger than 14 and 21 d old at the time of DT injection, respectively (Fig. 2*D*,*E*; for details in estimation, see Materials and Methods), indicating that most ablated neurons were at the early maturational stage.

Next, to characterize the time course of neuronal ablation, we injected the viral vector into one hemisphere of the dentate gyrus and administered DT one week later (Fig. 3*A*). Either 1, 4, or 7 d after the DT injection, we analyzed DCX+ cell density. The density in the injected hemispheres were normalized by dividing by the density in non-injected hemispheres in the same mice. Normalized DCX+ cell density showed a significant, negative correlation with days after DT injection (*r* = −0.843, *p* = 5.7 × 10^−4^, *n* = 12 mice; Fig. 3*B*,*C*), indicating that normalized DCX+ cell density reduced over time. Further we performed two-way (hemisphere × day) repeated measures ANOVA, and detected significance in the main effect of day and hemisphere and hemisphere × day interaction (day: *p* = 0.0023, *F*_(2,9)_ = 12.869, hemisphere: *p* = 2.0 × 10^−5^, *F*_(1,9)_ = 65.419, hemisphere × day: *p* = 0.0023, *F*_(2,9)_ = 12.869, *n* = 12 mice in total). One sample *t* test against 1 indicated that the normalized density of DCX+ cells were significantly reduced in injected hemispheres than in non-injected controls at all three time points (1 d: *p* = 3.0 × 10^−4^, *t*_(3)_ = −19.484, 4 d: *p* = 0.016, *t*_(3)_ = −4.906, 7 d: *p* = 0.011, *t*_(3)_ = −5.573). Independent sample *t* test between days showed that the normalized density at 4 d after DT injection was not significantly lower than that at 1 d (*p* = 0.062, *t*_(3.064)_ = 2.880, independent *t* test), but reached significance at 7 d (*p* = 0.020, *t*_(3.016)_ = 4.548). Thus, the ablation of immature neurons gradually occurred over multiple days.

Most ablation methods used in similar studies induces inflammation-like response (Arruda-Carvalho et al., 2011; Seo et al., 2015). Therefore, we quantified the number of Iba1+ cells in the dentate gyrus, whose increase indicates inflammation-like response. An increase in the number of Iba1+ cells was detected after the induction of ablation by a combination of viral vector and DT injections (Fig. 4). It is known that inflammation can cause a detrimental effect on brain functions including memory, which is associated with damages in neuronal structures (DiSabato et al., 2016). We found that the volume (Figs. 1*H*, 5*A*,*B*; quantitative results of larger cohorts in Fig. 6*D*, 7*E*, 8*C*) and cell density (Fig. 5*B*,*C*) of the granule cell layer remained intact. The morphology of mature granule cells (Fig. 5*D–G*) was also unaffected. These observations indicate that DT injection ablated DCX+ cells without inducing the large-scale elimination or damage of mature neurons in the granule cell layer. We found that the ablation technique does not affect neurogenesis either in the olfactory bulb or in the subventricular zone (Fig. 5*H*).

In the experiment in which we examined the time course of ablation, we also assessed the potential non-specific effects of ablation on the activity of other neurons in the granule cell layer. We analyzed the expression of an immediate early gene product, c-fos protein, as a proxy for protein synthesis and neuronal activation (Fig. 3*D*,*E*). To induce c-fos expression, the mice were exposed to a novel environment 90 min before perfusion. Two-way (hemisphere × day) repeated measures ANOVA did not detect any significance in the main effects or interaction (day: *p* = 0.613, *F*_(2,9)_ = 0.516, hemisphere: *p* = 0.772, *F*_(1,9)_ = 0.090, hemisphere × day: *p* = 0.613, *F*_(2,9)_ = 0.516). While sample number is limited, c-fos protein expression was similar between injected and control hemispheres (95% confidence interval of difference in hemisphere effect, [−0.011, 0.066]; in day 1, [−0.251, 0.131]; in day 4, [−0.138, 0.210]; in day 7, [−0.253, 0.225]). In addition, we did not detect significant correlation between the normalized c-fos+ cell density and days after DT injection (*r* = 0.144, *p* = 0.654, 95% confidence interval of *r*, [−0.469, 0.663]). Thus, using limited number of samples, we did not detect any clear structural and functional sign of damages which inflammation may have caused.

### Postlearning ablation impaired the persistent search for missing platform in probe tests of a water maze task

Next, we induced the ablation after task training in a water maze task and examined its effects on their performance in a probe test (Fig. 6*A*). First, we pretrained the mice over 4 d using a visible platform to familiarize them with the water maze procedures (pretraining). The mice were bilaterally injected in the dentate gyrus with the viral vector. After a 7-d recovery period, the mice were trained in a hippocampus-dependent water maze task using a hidden platform in a constant position over 24 trials in 1 d (training; Trouche et al., 2009). At the completion of the 24 trials, DT was systemically administered to the mice to induce the ablation (ablated group). Seven days after training, probe tests were performed to evaluate memory for the position where the platform was located during training. These tests were followed by re-training with new platform positions each day over 3 d. The control group were trained and tested in the same protocol except ablation was not induced; a half of control group was injected with vehicle instead of the viral vector and the other half was injected with vehicle instead of DT. We confirmed a reduction of DCX+ cells in the ablated group (*p* = 0.011, *t*_(31.241)_ = 2.688, *n* = 18 mice for each group, independent sample *t* test; Fig. 6*B*,*C*), while the volume of the granule cell layer was intact (*p* = 0.939, *t*_(34)_ = 0.077, independent sample *t* test; Fig. 6*B*,*D*). In pretraining and training (which were conducted before the ablation was induced), both groups learned the platform positions similarly well (Fig. 6*E*,*F*; Table 1).

**Table 1:** Statistical results for **Figure 6**

Pretraining (three-way, group × trial × day, control, *n* = 18 mice; ablated, *n* = 18 mice)						
	Latency to platform			Speed		
	*p*	df	*F*	*p*	df	*F*
						
Day	0.016	3, 102	3.607	6.5 × 10^−7^	2.434, 82.753	14.743
Day × group	0.858	3, 102	0.254	0.630	2.434, 82.753	0.524
Trial	1.4 × 10^−11^	1.529, 51.982	52.033	0.006	2, 68	5.494
Trial × group	0.885	1.529, 51.982	0.072	0.612	2, 68	0.495
Day × trial	0.459	4.993, 169.754	0.936	0.007	6, 204	3.023
Day × trial × group	0.713	4.993, 169.754	0.583	0.226	6, 204	1.375
Group	0.870	1, 34	0.027	0.157	1, 34	2.089
Training (two-way, block × group, control, *n* = 18 mice; ablated, *n* = 18 mice)						
	Latency to platform			Speed		
	*p*	df	*F*	*p*	df	*F*
Block	2.6 × 10^−12^	4.313, 146.653	17.434	7.8 × 10^−5^	5.291, 179.911	5.440
Block × group	0.321	4.313, 146.653	1.184	0.474	5.291, 179.911	0.919
Group	0.566	1, 34	0.336	0.533	1, 34	0.398
Re-training (three-way, group × trial × day, control, *n* = 18 mice; ablated, *n* = 18 mice)						
	Latency to platform			Speed		
	*p*	df	*F*	*p*	df	*F*
Day	0.034	2, 68	3.565	0.065	1.682, 57.174	3.020
Day × group	0.815	2, 68	0.205	0.826	1.682, 57.174	0.149
Trial	1.7 × 10^−30^	5.849, 198.874	38.729	0.0002	7, 238	4.199
Trial × group	0.756	5.849, 198.874	0.562	0.494	7, 238	0.917
Day × trial	0.478	9.888, 336.198	0.960	0.127	12.302, 418.255	1.479
Day × trial × group	0.871	9.888, 336.198	0.524	0.627	12.302, 418.255	0.826
Group	0.920	1, 34	0.010	0.265	1, 34	1.282

For the probe tests, we analyzed (1) the time that the mice spent in proximity to the platform and (2) the number of entries into the area where the platform was located (Fig. 6*G*,*H*). The ablated group (*n* = 18 mice) showed significantly lower values in both parameters than the control group (*n* = 17 mice; *p* = 0.008, *t*_(27.035)_ = 2.877 and *p* = 0.019, *t*_(33)_ = 2.465, respectively, independent-sample *t* tests; Fig. 6*I*), whereas both control and ablated groups spent significantly longer time near platform than the chance level (4.3 s; control: *p* = 2 × 10^−6^, *t*_(16)_ = 7.244, ablated: *p* = 1.1 × 10^−5^, *t*_(17)_ = 6.151, one-sample *t* test; Fig. 6*I*). These results indicate that, while both groups remembered the platform position, the ablated group showed a deficit in memory retrieval.

To further examine the details in pattern of the deficit, we quantified other parameters. Unexpectedly, latency to reach former platform area was comparable between the two groups (*p* = 0.922, *t*_(33)_ = −0.099, independent-sample *t* test; Fig. 6*J*), suggesting that performance impairment occurred specifically after the mice reached the former platform area. This is why we focused on the period after the first entry to the platform position. Similar to the whole-duration analysis in Figure 6*I*, the proportion of time in which the mice spent near platform and the number of entries into the former platform area per unit time were significantly lower in the ablated group than the control group (*p* = 0.0062, *t*_(23.352)_ = 3.008 and *p* = 0.013, *t*_(33)_ = 2.615, respectively, independent-sample *t* tests; Fig. 6*K*). These results indicate that memory retrieval was initially intact in the ablated group and as accurate as the control group; after initial failure to find the platform the control group persisted to search platform while the ablated group reduced search prematurely. In addition, we found that the duration of individual visits near platform was shorter in the ablated group (*p* = 0.022, *t*_(20.741)_ = 2.469, independent-sample *t* test; Fig. 6*L*). Although the time scale is different, this result also indicates that persistence of platform search was impaired in the ablated group. Swimming speed was not significantly different between the groups (*p* = 0.884, *t*_(33)_ = 0.148, independent-sample *t* test; Fig. 6*I*). In re-training, both groups learned the platform positions similarly well (Fig. 6*M*; Table 1).

### Postlearning ablation impaired the persistence of tone-induced freezing behavior after tone trace fear conditioning

To test whether the effect of the postlearning ablation on persistence of learned behavior is generalized to another form of hippocampus-dependent memory, we examined the effect of the postlearning ablation on a trace fear conditioning task. Similarly, to the water maze experiment, one week after virus injection, mice received training for trace fear conditioning (Fig. 7*A*). The mice were exposed to five pairings of a tone (a conditioned stimulus) and an electrical shock (an unconditioned stimulus) with a 20-s interval in context A (Fig. 7*B*). After the completion of training, mice in the control and ablated groups (*n* = 13 and 12 mice, respectively) received systemic injection of PBS and DT, respectively. The two groups showed similar freezing level during training (which was conducted before inducing the ablation) both before and after the first tone was given (*p* = 0.471, *t*_(23)_ = 0.733 and *p* = 0.643, *t*_(23)_ = −0.470, respectively, independent-sample *t* tests; Fig. 7*C*). We confirmed a reduction of DCX+ cells in the ablated group (*p* = 3 × 10^−6^, *t*_(15.705)_ = 7.096, independent sample *t* test; Fig. 7*D*) while the volume of the granule cell layer was intact (*p* = 0.556, *t*_(23)_ = 0.597, independent sample *t* test; Fig. 7*E*).

One week after training, mice underwent a tone test in context B, in which mice were exposed to five tones without a foot shock (Fig. 7*F*,*G*). We analyzed percentage time in freezing during 180 s baseline period (before first tone exposure) and five consecutive 20-s periods (tone, posttones 1, 2, 3, and 4) averaged over five tone exposures (Fig. 7*F*,*H*). Two-way (period × group) repeated measures ANOVA detected significant effects of period and group and significant interaction between period and group (period: *p* = 2.0 × 10^−22^, *F*_(4.242,115)_ = 46.378, group: *p* = 0.021, *F*_(1,23)_ = 6.117, period × group: *p* = 0.002, *F*_(4.242,115)_ = 4.576; Fig. 7*H*). The two groups showed comparable level of freezing during the baseline period and similarly increased freezing level in Tone period (*p* = 0.252, *t*_(23)_ = 1.175, and *p* = 0.926, *t*_(23)_ = 0.094, respectively, independent-sample *t* tests). However, from posttone 1 onwards, freezing level of the ablated group became lower than the control group, which became significant during posttone 3 and 4 periods (posttone 1: *p* = 0.055, *t*_(23)_ = 2.021, posttone 2: *p* = 0.081, *t*_(23)_ = 1.828, posttone 3: *p* = 0.001, *t*_(20.222)_ = 3.755, posttone 4: *p* = 0.017, *t*_(18.333)_ = 2.618, independent-sample *t* tests). When we focused on the response to the first tone exposure only (instead of average over 5 exposures), the ablated group showed a similar reduction specifically at later time points (period: *p* = 5.6 × 10^−14^, *F*_(5,115)_ = 19.419, group: *p* = 0.064, *F*_(1,23)_ = 3.786, period × group: *p* = 4.5 × 10^−5^, *F*_(5,115)_ = 6.128, two-way repeated measures ANOVA; baseline: 0.252, *t*_(23)_ = 1.175, tone: *p* = 0.404, *t*_(23)_ = −0.851, posttone 1, 2, 3, 4: *p* = 0.522, 0.139, 8.4 × 10^−4^, 0.014, *t*_(23)_ = 0.651, 1.532, 3.839, 2.662, respectively, independent-sample *t* tests; Fig. 7*I*). Thus, freezing response of the ablated group was initially intact and increased similarly to the control group. However, this increase did not persist as long as it did in the control group.

We have also analyzed the frequency and duration of individual freezing episodes. The frequency of freezing episodes did not show a significant difference between the two groups and similarly increased from baseline to after the first tone exposure (period: *p* = 3.2 × 10^−7^, *F*_(1,23)_ = 50.200, group: *p* = 0.743, *F*_(1,23)_ = 0.110, period × group: *p* = 0.097, *F*_(1,23)_ = 2.990, two-way repeated measures ANOVA; Fig. 7*J*). For the duration of freezing episodes (Fig. 7*K*), two-way repeated measures ANOVA (period × group, control: *n* = 13 mice, ablated: *n* = 12) detected a significant effect of period (*p* = 4.5 × 10^−8^, *F*_(1,23)_ = 63.568) and group (*p* = 0.029, *F*_(1,23)_ = 5.427) and significant interaction (period × group: *p* = 0.017, *F*_(1,23)_ = 6.571). In the baseline period, the duration was comparable between the groups (*p* = 0.356, *t*_(23)_ = 0.942, independent-sample *t* test). In contrast, after the onset of first tone, the duration was significantly shorter in the ablated group than in the control group (*p* = 0.019, *t*_(16.486)_ = 2.600, independent-sample *t* test). Thus, the persistence of individual freezing episodes was impaired by the postlearning ablation.

### Postlearning ablation impaired the persistence of context-induced freezing behavior after contextual fear conditioning

Next, we examined whether the postlearning ablation affects contextual fear conditioning, another form of hippocampus-dependent fear conditioning paradigm (Fig. 8*A*). 7 d after viral vector injection, the mice underwent training for contextual fear conditioning. The mice were moved to context A and an electrical shock (2 s) was given after 180 s (training). Next day, a shorter shock (0.2 s) was given as a reminder in the same context (“reminder” training), and then DT was systemically injected (ablated group, *n* = 18 mice). After 7 d, context test was conducted. The control group (*n* = 20 mice) underwent the same protocol except they were injected with viral vector+PBS or PBS+DT. We confirmed a reduction of DCX+ cells in the ablated group (*p* = 7 × 10^−6^, *t*_(31.165)_ = 5.363, independent sample *t* test; Fig. 8*B*), while the volume of the granule cell layer was intact (*p* = 0.768, *t*_(36)_ = −0.298, independent sample *t* test; Fig. 8*C*). In training and reminder training (which were conducted before inducing the ablation), we did not find significant difference between the two groups in freezing level before or after the foot shock [baseline: *p* = 0.700, *t*_(36)_ = −0.389, after shock: *p* = 0.568, *t*_(36)_ = 0.576 (Fig. 8*D*); baseline: *p* = 0.719, *t*_(36)_ = −0.363, after shock: *p* = 0.455, *t*_(36)_ = 0.755, independent-sample *t* tests (Fig. 8*E*)].

In the context test, overall freezing level was significantly lower in the ablated group compared with control (*p* = 0.046, *t*_(33.9)_ = −2.074, independent-sample *t* test; Fig. 8*F*,*G*), indicating that the postlearning ablation impaired fear memory. Motivated from the previous two experiments showing impairment at late, but not early, time points in memory retrieval tests, we examined (1) latency for cumulative freezing duration to reach 20 s; (2) percentage of time in freezing before reaching cumulative freezing duration of 20 s; and (3) the same percentage after reaching cumulative freezing of 20 s. Cumulative duration of 20 s is an arbitrary value which corresponds to ∼20% of mean total freezing time. We did not find significant group difference in the first two, which are the indexes of memory retrieval performance at early time points in the test (*p* = 0.622, *t*_(36)_ = −0.963 and *p* = 0.322, *t*_(31.164)_ = 1.006, independent-sample *t* tests; Fig. 8*H*,*I*). In contrast, we found a significant reduction in percentage time in freezing after cumulative freezing duration reached 20 s (*p* = 0.033, *t*_(36)_ = 2.216, independent-sample *t* test; Fig. 8*J*). We have also analyzed the number and duration of freezing episodes. Consistent with tone fear conditioning, the number of freezing episodes was not different between the groups (*p* = 0.285, *t*_(36)_ = −1.086, independent-sample *t* test; Fig. 8*K*), but their duration was significantly shorter in the ablated group than control (*p* = 0.028, *t*_(27.742)_ = 2.323, independent-sample *t* test). Thus, the persistence of learned fear responses to the context was impaired by the posttraining ablation.

## Discussion

### Methodological consideration

We established a novel method ablating DCX+ immature neurons in the dentate gyrus. Ablation is highly selective to immature neurons at the early maturational stages; >90 and >95% of ablated neurons are estimated to be less than three and less than four weeks old, respectively. However, the method causes a side effect of inflammation-like response detected by increase in Iba1+ cells. Similar inflammation-like response has been also observed in previous studies that used different methods involving cell ablation (Arruda-Carvalho et al., 2011; Seo et al., 2015). Inflammation is known to cause damages in neurons and compromise their functions (DiSabato et al., 2016). Therefore, we cannot distinguish whether the observed behavioral change is because of inflammation or ablation of immature neurons. We have evaluated a sign of detrimental effects on other neuronal populations than DCX+ immature neurons in the dentate gyrus. However, we did not detect any structural sign of damages in granule cell layer volume, the density, dendritic complexity and spine density of granule cells. Functionally, we did not detect a clear change in c-fos expression, a proxy of neuronal activation. Intact memory performance in re-training for new platform positions after ablation suggests that the overall function of dentate gyrus is intact (Sutherland et al., 1983; Nanry et al., 1989).

### Main finding: impairment in within-trial persistence of learned behavior and its dissociation from preserved performance in the initial phase of retrieval tests

In this study, we showed that the postlearning ablation results in behavioral deficits during memory retrieval tests of three hippocampus-dependent memory tasks. In these widely used retrieval tests, animals respond to cues (i.e., visual cues, context, tone) and show learned behavior (platform search and freezing). However, the learned behavior is not reinforced because of the absence of the platform or electrical shocks. Although the absence of reinforcement eventually leads to reduction/cessation of learned behavior [so called “extinction” or “within-session extinction” (Myers and Davis, 2002). In this context, in the meaning at the behavioral level as often used in psychology], animals continue to show the learned behavior for some duration, which we term as “persistence of learned behavior” (note that we use the term “persistence” as a description of behavior but not cognitive ability/tendency). We observed that the postlearning ablation impairs such persistence of learned behavior, which contains two common features. First, the ablated group showed intact performance of learned behavior at early time points during the memory retrieval tests. However, later in the tests, the ablated group reduced learned behavior compared with control. Second, when we examined the duration of individual episodes of learned behavior (platform search and freezing), their durations were reduced in the ablated group. Both of these common features point to impairment in persistence of learned behavior, although they are different in their timescales, over the entire trials and for individual behavioral episodes. These effects were consistently observed in three hippocampus-dependent memory tasks.

We found that the effect of the postlearning ablation showed dissociation between initial performance and persistence of learned behavior; the ablation impaired persistence while preserving initial performance intact. In typical studies, it is rare for these measurements to be considered separately in memory retrieval tests, and rather expected to be strongly correlated with each other. Although previous studies separated different behavioral components in a single type of tasks (Dalm et al., 2000; Korz, 2006; Maei et al., 2009), it is rare to make an attempt to find such dissociable behavioral components that are common across different types of hippocampus-dependent memory tasks with different behavioral and cognitive demands. We have found the dissociation consistently in the three hippocampus-dependent tasks.

The observed dissociation effect indicates that there are at least two separate components of behavior during the memory retrieval tests. These two components, initial performance and persistence, are at least partially independent and dissociable in the sense that the latter can be high or low even if the former is similarly well. Furthermore, the dissociated effect suggests that two separate brain functions and their underlying mechanisms govern learned behavior in the memory retrieval tests. One function supports persistence of learned behavior, which was impaired by the ablation. The other supports initial expression of learned behavior, which was not affected by the postlearning ablation.

### Speculating about relationship between persistence of learned behavior and memory strength

In this section, we speculate about relationship between persistence of learned behavior and memory. In typical behavioral studies on mechanisms underlying memory, one interprets a decrease in expression of learned behavior caused by an experimental manipulation as weakening/impairment of memory while an increase as strengthening/improvement of memory. We found that our postlearning manipulation impairs performance specifically at late time points of memory retrieval tests, but not that at early time points. Applying the same interpretation method as above, we could interpret our finding as an indication that the postlearning manipulation impairs the aspect of memory strength that regulates behavior at late time points while sparing another aspect of memory strength that influences behavior at early time points. This interpretation of dissociated effects between the early and late time points also hints at or assumes an idea that there may be two separate aspects of memory strength supporting learned behavior at different time points, which have separate underlying mechanisms.

Expression of learned behavior is thought to depend on memory storage and retrieval. For example, recent studies indicated that amnesia induced by protein-synthesis inhibitor or in mouse models of Alzheimer’s disease is because of compromised memory retrieval but not storage (Ryan et al., 2015; Roy et al., 2016), which may indicate that the strength/effectiveness of these two processes may be independent and separable. Bjork and Bjork postulated two types of memory strength (Bjork and Bjork, 1992); one refers to how easily the learned information is retrieved while the other represents how well/detailed learned information is retained. The latter may be related to vividness of or subjective certainty about a memory, which could influence persistence of learned behavior. Imagine that you vividly remember that you stored your room key in a drawer of a cabinet last night and you are 100% sure about it. Even if your initial search attempt failed to find the key in the drawer, you would continue to search the key for a long time. On the other hand, if you are not sure about where you stored the key and happen to search the drawer, you would stop it more quickly and start searching other locations. Studies have showed that human subjects made a more persistent effort in searching a missing object or recalling a difficult-to-be-retrieved memory when they were more certain about the memories (Gruneberg et al., 1977; DeLoache and Brown, 1984). Although we cannot tell what exactly is reflected by the mouse behavior, it would be reasonable to speculate that initial performance and persistence of learned behavior in memory retrieval tests reflect different aspects of memory in terms of how well a mouse remember the memory.

So far in this section, we discussed that persistence of learned behavior may reflect memory strength, which is a characteristic of individual memories. However, an alternative way to explain our observation is that ablation impaired subject’s cognitive ability/tendency of translating learned information into long-lasting behavior within a trial. For example, cognitive stability is the ability to keep one’s perspective stable without being affected by environmental changes/distractions (Ueltzhöffer et al., 2015; Dreisbach and Fröber, 2019; Uddin, 2021), in other words, the ability to suppress cognitive flexibility or, in memory-related contexts, the ability to keep long-lasting working memory. Disruption of within-trial cognitive stability would result in premature cessation of subject’s perspective based on learned information, which can explain our observation of reduction in learned behavior at late phases in memory retrieval tests.

How can we distinguish whether observed changes in within-trial persistence of learned behavior represents impairment in specific memories or cognitive ability/tendency? If impairment is specific to a subset of memories, we would observe changes in within-trial persistence for some memories but not for others. On the other hand, if impairment is in cognitive ability/tendency, we would observe changes in within-trial persistence similarly for all memories. Starting from 1 d after the water maze probe tests, we performed retraining for 3 d while changing platform location each day. By the end of days 1 and 2, mice would have learned new platform locations well. In the next trials (the first trials of days 2 and 3, respectively), mice experienced similar situations to probe tests where the platform was not located at the newly learned locations. In these trials, how persistently mice continue to search the former platform positions (learned on days 1 and 2, respectively) would delay mice to reach the platform in a new position. If cognitive ability/tendency was affected, we should have seen a change in performance in these trials. If impairment was specific to the memory for original platform position during training, such a change would not be expected. Our result is consistent with the latter possibility, although our retraining experiment may not necessarily be optimized to address this question and we need further investigation to reach a firm conclusion. In addition, distinction between the possibilities may not be straightforward purely based on behavioral observation and would require further studies involving different techniques.

### Potential involvement of DCX+ immature neurons in persistence of learned behavior

We observed that the postlearning ablation of immature neurons caused inflammation-like response and therefore are not able to attribute the observed impairment in persistence to the ablation of DCX+ immature neurons. Nonetheless, we believe it is worth discussing existing literature both consistent and seemingly conflicting with our results from the viewpoint of a potential postlearning role of immature neurons. First, previous studies demonstrated a role of immature neurons at the early maturational stage in memory by removing immature neurons before memory formation (Deng et al., 2009; Seo et al., 2015). These findings are consistent with our finding, but do not differentiate whether those immature neurons are involved in initial memory acquisition only or later memory processes because they blocked adult neurogenesis before memory acquisition. Second, other studies found memory impairment when ablating adult-born neurons (Arruda-Carvalho et al., 2011; Suárez-Pereira et al., 2015) or silencing them (Kumar et al., 2020) after training for hippocampus-dependent tasks. These findings are consistent with our finding in that new neurons have a role beyond initial memory acquisition, although they did not reveal the age of new neurons involved in this role. Third, using optogenetic silencing during memory retrieval tests, Gu et al. (2012) indicated that the postlearning role is mediated by new neurons at later maturational stages, but failed to detect impairment when DCX+ immature neurons was silenced during memory retrieval tests. At a quick look, one may find this finding conflicting with ours. However, they performed optogenetic silencing only during memory retrieval tests while ablation in our method was induced immediately after the completion of training. Therefore, it is possible that the existence of new neurons at early maturational periods is required during memory retention period but not during memory retrieval. Fourth, Vukovic et al. (2013) performed a conceptually similar experiment involving immature neuron ablation using an active place avoidance task. This study did not find impaired performance in retrieval test trials when immature neurons were ablated after normal acquisition of a place avoidance task. An important difference from our study in the experimental design is the presence of reinforcement during memory retrieval tests. During training for the active place avoidance task, mice learned to stay in a safe zone to avoid noxious electrical shocks; otherwise, they received electrical shocks when they went out of the safe zone. This reinforcing design was kept same in the memory retrieval tests, so that the learned behavior continued to be reinforced. Therefore, the retrieval test itself supported persistence of learned behavior and may have masked the contribution of DCX+ immature neurons being reflected to persistent behavior. This may be why this previous study did not find an effect after postlearning ablation of DCX+ immature neurons.

New neurons at the early immature stage are in the process of constructing new circuits by extending neurites, forming synapses and making survival-or-death decisions (Biebl et al., 2000; Zhao et al., 2006). These phenomena are experience/activity dependent (Ge et al., 2006; Overstreet-Wadiche et al., 2006; Tashiro et al., 2006, 2007; Tronel et al., 2010; Chancey et al., 2013; Aasebø et al., 2018). Further, although long-term potentiation is more easily induced in immature neurons at the early stage than in mature neurons (Wang et al., 2000; Snyder et al., 2001; Schmidt-Hieber et al., 2004), the extent of the potentiation is smaller in younger neurons than in their older counterparts (Ge et al., 2007). These characteristics seem to better support gradual changes which do not complete immediately during memory acquisition but accumulate over time. Through these slow circuit modification processes, DCX+ immature neurons may influence how acquired memory supports the expression of learned behavior and therefore their presence during memory retention period is required to form strong memory that leads to persistent learned behavior. A further study using a postlearning manipulation without inflammatory and other side effects would be required to determine whether DCX+ immature neurons have a postlearning role in persistence of learned behavior.

In conclusion, our work revealed that there are dissociable behavioral components during memory retrieval tests of hippocampus-dependent memory tasks, which have been widely used in rodents. These two behavioral components, initial performance and persistence of learned behavior, are likely to reflect different brain functions and be mediated by separate mechanisms, which might represent different modalities of memory strength. These simple dissociable measurements in widely used behavioral paradigms would be useful to understand the detailed mechanisms underlying the expression of learned behavior and potentially different modalities of memory strength in mice.

## Synthesis

Reviewing Editor: Erica Glasper, University of Maryland Decisions are customarily a result of the Reviewing Editor and the peer reviewers coming together and discussing their recommendations until a consensus is reached. When revisions are invited, a fact-based synthesis statement explaining their decision and outlining what is needed to prepare a revision will be listed below. The following reviewer(s) agreed to reveal their identity: Jake Jordan. Note: If this manuscript was transferred from JNeurosci and a decision was made to accept the manuscript without peer review, a brief statement to this effect will instead be what is listed below.

Manuscript Name: Within-trial persistence of learned behavior as a dissociable behavioral component in conventional hippocampus-dependent memory tasks: a potential post-learning role of immature neurons in the adult dentate gyrus

Manuscript Number: eN-TNWR-0195-21X

Synthesis: The authors present a novel method used to permanently ablate immature neurons in the dentate gyrus of the hippocampus. Data presented in the manuscript demonstrate, via the use of several hippocampal-dependent tasks, that ablated mice have preserved initial performance but deficits in perseverance of learned behavior. There is consensus that this work is important as the ability to measure independent modalities of memory strength during behavior, using common memory tasks, would very helpful to the field. believe the work is worthy of publication and is novel, helpful, and important for our field. While the work is promising, it is recommended that the manuscript be revised prior to submission, but only requiring additional review from the reviewing editor. The major concerns are as follows and phrases in quotes are directly from the individual reviewers:

1.Overall, the introduction and discussion should be reoriented and expanded to provide even emphasis on behaviors underlying memory and adult neurogenesis. “There is no mention of adult neurogenesis despite ablation being the main manipulation; a rationale of why adult born neurons are important for this topic is needed. Additionally, a more in-depth discussion of memory measures, such as a definition of persistence, would be helpful.”

2.The disjointedness may be a result of the revision that has already taken place.

3.Along the same lines, given the revisions that this manuscript has received, there appears to be a disconnect between the stated aim of the paper and the content of the figures. Some of the control data may present better in supplementary.

4.The authors are encouraged to consider a different interpretation of the “within trial persistence” behavior. It does not appear from this manuscript alone that this is actually being measured. Could impairment of persistence be what is measured or a separate cognitive process, such as cognitive flexibility? Alternative explanations (e.g. cognitive flexibility) need to be discussed as well.

5.While persistent inflammation does not affect c-fos response to the open field task, it does not rule out changes to physiology of granule cells in the hippocampus. It is recommended that the authors be “straightforward and clear” in the discussion that it is possible that behavioral changes may be due to inflammation or ablation of immature neurons, and the current study cannot say ascertain which. While the authors hint at this, stronger language may be appropriate here.

6.Although the results and conclusions are focused on the behavioral data, the majority of the figures are related to the ablation methods and controls. Specifically, the c-Fos experiment in the manuscript needs to include a power analysis or justification of the appropriate sample size or experiments as they are. It is suggested that the c-fos data be removed from the figures and made in text only followed by a cautious statement such as “using a limited sample of tissue, c-fos protein expression was similar between injected and sham hemispheres, suggesting...”.

Minor concerns that should be considered in the revised manuscript are:

1.The title should be more succinct.

2.Please define acronyms on first use (such as DTR, DCX, etc.)

3.Please justify the use of only female mice for the behavior tasks, even though both male and female mice are used in ablation figures.

4.Quantifications are probably sufficient to show the DCX reduction for each behavior experiment. Representative images are not needed each time. Remove figures 7-8 (or making them smaller/

## Author Response

Synthesis: The authors present a novel method used to permanently ablate immature

neurons in the dentate gyrus of the hippocampus. Data presented in the manuscript

demonstrate, via the use of several hippocampal-dependent tasks, that ablated mice

have preserved initial performance but deficits in perseverance of learned behavior.

There is consensus that this work is important as the ability to measure independent

modalities of memory strength during behavior, using common memory tasks, would

very helpful to the field. believe the work is worthy of publication and is novel, helpful,

and important for our field.

We would like to thank the editor and reviewers for evaluating our manuscript. We are

happy to head about your positive evaluation of our work.

While the work is promising, it is recommended that the manuscript be revised prior to

submission, but only requiring additional review from the reviewing editor. The major

concerns are as follows and phrases in quotes are directly from the individual reviewers:

1. Overall, the introduction and discussion should be reoriented and expanded to

provide even emphasis on behaviors underlying memory and adult neurogenesis. “There

is no mention of adult neurogenesis despite ablation being the main manipulation; a

rationale of why adult born neurons are important for this topic is needed. Additionally,

a more in-depth discussion of memory measures, such as a definition of persistence,

would be helpful.”

Regarding introduction, we moved the first paragraph of the Result section to the

introduction section. This paragraph about adult neurogenesis was a part of the

introduction section in the previous version of manuscript that we submitted elsewhere

before eNeuro. Upon the change in the main points of the manuscript, we previously

moved this paragraph to the Result section. Following the editor/reviewers’ advice, we

now moved it back to the introduction section.

We believe that the discussion section covers the two topics more or less evenly. We

added a paragraph discussing alternative interpretation about cognitive stability at Ln

649-664. To address the specific comment about the definition of persistence, we added

the definition at Ln 591-593.

2. The disjointedness may be a result of the revision that has already taken place.

Yes. We believe that this is true. The editor/reviewers’ comment helped us improve our

manuscript in this respect.

3. Along the same lines, given the revisions that this manuscript has received, there

appears to be a disconnect between the stated aim of the paper and the content of the

figures. Some of the control data may present better in supplementary.

We agree that some of our figures may suit as supplementary figures. However, because 2

eNeuro does not allow authors to include supplementary figures, we presented them as

main figures in the original manuscript. Following the editor/reviewers’ comment, we

checked the journal’s policy carefully again. We found that eNeuro allows authors to

publish Extended Data. However, our Figure does not fit to the nature of Extended Data

according to Editor-in-Chief’s commentary (Bernard, eNeuro, 4: 0103-17, 2017).

Therefore, we kept them as main figures.

4. The authors are encouraged to consider a different interpretation of the “within trial

persistence” behavior. It does not appear from this manuscript alone that this is actually

being measured. Could impairment of persistence be what is measured or a separate

cognitive process, such as cognitive flexibility? Alternative explanations (e.g. cognitive

flexibility) need to be discussed as well.

As we inquired through email, this editor/reviewers’ comment is unclear to us. As we did

not get a reply, we revised the manuscript based on our best guess, which we believe is

correct understanding of the comment.

First of all, we want to make sure that, in this manuscript, we use the term “persistence"

purely as a description of behaviour, but not of cognitive ability/tendency. We now

clearly stated this at Ln 591-593.

In the original manuscript, our discussion focused on memory strength. Following the

editor/reviewers’ recommendation, we added two paragraphs in Discussion section at Ln

653-680. As we observed that ablation reduce behavioural persistence, we focused on

cognitive stability (opposite to cognitive flexibility), which may be disrupted by ablation.

5. While persistent inflammation does not affect c-fos response to the open field task, it

does not rule out changes to physiology of granule cells in the hippocampus. It is

recommended that the authors be “straightforward and clear” in the discussion that it is

possible that behavioral changes may be due to inflammation or ablation of immature

neurons, and the current study cannot say ascertain which. While the authors hint at this,

stronger language may be appropriate here.

We agree with the editor/reviewers, and more clearly state the limitation of our study as

below at Ln 572-574.

"Therefore, we cannot distinguish whether the observed behavioral change is due to

inflammation or ablation of immature neurons.”

6

. Although the results and conclusions are focused on the behavioral data, the majority

of the figures are related to the ablation methods and controls. Specifically, the c-Fos

experiment in the manuscript needs to include a power analysis or justification of the 3

appropriate sample size or experiments as they are. It is suggested that the c-fos data be

removed from the figures and made in text only followed by a cautious statement such

as “using a limited sample of tissue, c-fos protein expression was similar between

injected and sham hemispheres, suggesting...”.

We agree with the editor/reviewers that the non-significant effect of the c-fos

experiment should not be interpreted as there are no effect on c-fos expression as it is

often (improperly) done in many other studies. We regret that we previously made

strong statements in Results and Discussion sections. As the editor/reviewers suggested,

we revised to a cautious statement at Ln 423-427 and 577.

Regarding the suggestion for a power analysis, we would like the editor/reviewers to

note that the use of post-hoc power analyses or observed power for evaluating nonsignificant effect has been criticized as a wrong practice with conceptual flaws (Hoenig

and Heisey, The American Statistician, 55: 19-24, 2001,

https://doi.org/10.1198/000313001300339897; Zumbo and Hubley, The Statistician, 47:

385-388, 1998, https://doi.org/10.1111/1467-9884.00139; Zhang et al., Biostatistical

methods in psychiatry, 32: e100069, 2019, http://dx.doi.org/10.1136/gpsych-2019-

100069). The benefit of a power analysis for experimental design is widely accepted but

the use of a post-hoc analysis or observed power have been strongly discouraged.

Therefore, instead of observed power, we added confidence intervals at Ln 424-5 and

429 as eNeuro generally recommends as a part of estimation statistics advocacy. 95%

confidence interval shows how reliable the observed mean values is.

Regarding the figure, while we seriously considered to remove Figure 3D, E, we noted

that it is difficult to explain the result without using the image and graph. We believe

that, without the figure, readers would have a difficulty in understanding what we try to

communicate. We hope that the editor/reviewers allows us to keep the figure to avoid

producing a problem in readers understanding our work. We consider that clear

communication is one of the most important factor when we decide what to present as a

figure.

Minor concerns that should be considered in the revised manuscript are:

1. The title should be more succinct.

We agree that a shorter title is preferable. Indeed, before the original submission, we

spent a lot of time to think of a shorter title. However, we failed to come up with a

shorter title that represents all key points of our study. Therefore, we used the long title.

After reading the reviewer’s comment, we thought about a shorter title again. However,

we think we can only remove “conventional” and could not find a way to further make

the title shorter. Therefore, we decided to use “Within-trial persistence of learned

behavior as a dissociable behavioral component in hippocampus-dependent memory

tasks: a potential post-learning role of immature neurons in the adult dentate gyrus”.

This is within the eNeuro’s requirement for title length.4

2. Please define acronyms on first use (such as DTR, DCX, etc.)

Thank you for pointing out this shortcoming. We now defined them in their first use at

Ln 83, 84, 88.

3. Please justify the use of only female mice for the behavior tasks, even though both

male and female mice are used in ablation figures.

In initial characterization of the ablation method, we did not notice any clear sex

difference of ablation and performed general characterization without focusing sex

difference. For behavioural experiments, “to avoid behavioural variability between two

sexes” (added at Ln 108-109) from making it difficult to detect behavioural effects, we

used one sex only.

4. Quantifications are probably sufficient to show the DCX reduction for each behavior

experiment. Representative images are not needed each time. Remove figures 7-8 (or

making them smaller/

As the editor/reviewers suggested, we have removed the representative images from

Figure 7. (There are no representative images in Figure 8)
